# Impact of the COVID-19 pandemic on trends in health conditions associated with alcohol among patients with hypertension in Sweden

**DOI:** 10.1038/s41598-025-21712-0

**Published:** 2025-10-01

**Authors:** Marta A. Kisiel, Seika Lee, Jan Hasselström, Maria Hagströmer, Fredrik Nyberg, Mohammadhossein Hajiebrahimi, Axel C. Carlsson

**Affiliations:** 1 Occupational and Environmental Medicine, Department of Medical Sciences, Uppsala University, Uppsala, Sweden; 2 Occupational and Environmental Medicine, Uppsala University Hospital, Uppsala, Sweden; 3 Academic Primary Health Care Centre, Region Stockholm, Stockholm, Sweden; 4 Division of Family Medicine and Primary Care, Department of Neurobiology, Care Sciences and Society, Karolinska Institute, Huddinge, Sweden; 5 Division of Physiotherapy, Department of Neurobiology, Care Sciences and Society, Karolinska Institutet, Huddinge, Sweden; 6 School of Public Health and Community Medicine, Institute of Medicine, Sahlgrenska Academy, University of Gothenburg, Gothenburg, Sweden; 7 Pharmacoepidemiology and Social Pharmacy, Department of Pharmacy, Uppsala University, Uppsala, Sweden

**Keywords:** Alcohol-related disorders, Other alcohol-associated conditions, Hypertension, COVID-19, Pandemic, Cardiovascular disease, Time serries analysis

## Abstract

Research on how the COVID-19 pandemic, societal restrictions, and healthcare services barriers have impacted patients with hypertension is limited. This study aimed to evaluate trends in alcohol-related disorders, other alcohol-associated conditions, and deaths among patients with hypertension during the pandemic (March 2020–Feb 2022) compared to the pre-pandemic period (March 2018–Feb 2020) in Region Stockholm, Sweden. This exploratory descriptive time series analysis was conducted among adults diagnosed with hypertension between 2015 and 2018. Data were obtained from the Swedish National Patient Register (specialist care) and the Stockholm Region’s primary care database. The quarterly period prevalence of diagnoses or cumulative incidence of acute diagnoses and deaths was presented. The study included 168,963 patients with hypertension (57% females). Overall, no profound shifts in alcohol-related disorders or mortality were observed during the pandemic. However, noteworthy trends were: alcohol-related disorder diagnoses in primary care increased among females (3.2/1000 compared to 2.8–3.1/1000 pre-pandemic), while rates of alcohol dependency decreased in specialist care, particularly among males (3.5–4.1/1000 compared to 4.1–5.1/1000 pre-pandemic). Alcohol-related disorders and deaths remained higher in males than in females during both periods. Among other alcohol-associated conditions, cardiovascular disease prevalence increased in both sexes in primary care and in male patients in specialist care, whereas mental illness decreased in both sexes. This study highlights the need for continued prevention of hazardous alcohol use among patients with hypertension and monitoring of cardiovascular risk factors. Further research on hypertensive patients is needed, as the pandemic-related health impacts may not become apparent until many years later.

**Supplementary Information:**

The online version contains supplementary material available at 10.1038/s41598-025-21712-0.

## Introduction

Hypertension is one of the top causes of death worldwide ^1^. Between 1990 and 2019, the global number of people living with hypertension doubled from 650 million to 1.3 billion ^1^. High systolic blood pressure is associated with an annual burden of 235 million disability-adjusted life years (DALYs) lost ^2^. Despite its prevalence, hypertension often goes unrecognized: among adults aged 30–79 years with hypertension, only 54% are diagnosed, 42% receive treatment, and just 21% achieve well-controlled disease ^3,4^. Early detection and effective management are crucial for preventing stroke, chronic heart failure, atrial fibrillation, atherosclerosis, and dementia ^5^. Regular alcohol consumption is a significant risk factor for elevated blood pressure ^6^, with hazardous alcohol use being more common in hypertensive individuals than in the general population ^7^.

According to the World Health Organization (WHO), harmful alcohol consumption is linked to over 200 harmful health consequences, and is an estimated cause of 2.6 million deaths annually, two million of which occur in men ^8^. Hazardous alcohol consumption is estimated to contribute to 16% of hypertension cases globally ^9,10^. A dose-dependent relationship exists between alcohol intake, hypertension, and increased cardiovascular risk ^11^. Hazardous consumption of alcohol can also induce several direct health problems (in this study referred to as ‘’alcohol-related disorders’’), but is also associated with other conditions ranging from mental illness and cardio- and cerebrovascular diseases, to injury and accidents (in this study referred to as ‘’other alcohol-associated conditions’’) ^12^, as well as deaths due to suicides, poisoning and drowning ^13–15^.

The outbreak of severe acute respiratory syndrome coronavirus 2 (SARS-CoV-2), which causes coronavirus disease 2019 (COVID-19), was declared a global pandemic by the WHO in March 2020 ^16^. Early in the pandemic, concerns arose about potential shifts in substance use, particularly alcohol^17^. Several studies have investigated changes in alcohol consumption in the general population during this period. Meta-analyses indicate that while overall alcohol use decreased in Europe and Australia, it increased in the United States (US) compared to pre-pandemic levels ^18–20^. However, individuals with pre-existing high alcohol consumption showed intensified usage during the pandemic, as demonstrated in a longitudinal survey study in Norway ^21^. Only a few studies, all conducted in the US, have examined hospital admissions related to alcohol overuse during the pandemic. These studies found an increased prevalence of alcohol-related hepatitis leading to hospitalization ^22,23^. However, no research has specifically explored the impact of the pandemic on alcohol-related disorders among patients with hypertension.

Hazardous alcohol use has also been strongly linked to accidents, injuries, and suicides. Pre-pandemic meta-analyses show that alcohol use is associated with nearly doubled risk of suicide, and with an increased risk of drowning, poisoning, and accidental injuries. In addition, the pandemic may have not only influenced alcohol consumption, but also worsened existing health inequalities ^20^. Pandemic barriers to healthcare services and social isolation likely exacerbated these inequalities, leaving vulnerable patient groups, such as hypertension patients, without necessary support and at greater risk of alcohol-related harm ^24^.

The aim of this study was to evaluate the longitudinal trends of alcohol-related disorders and other selected alcohol-associated conditions, as well as deaths, in female and male adults with hypertension, during the COVID-19 pandemic in comparison to the pre-pandemic period. To capture a broad image of the issue, this study used diagnoses obtained in both primary care and specialist care in Region Stockholm, Sweden, applying descriptive time series analysis to capture and illustrate relevant trends over time.

## Materials and methods

### Study design and population

The present longitudinal descriptive explorative study used data from the SCIFI-PEARL (Swedish COVID-19 Investigation for Future Insights—a Population Epidemiology Approach using Register Linkage) project that includes regularly updated data from various national registers^25^. The prospectively collected data for specialist care came from the National Patient Register ^26^, and for primary care from the administrative health data register of Region Stockholm (including 2.2 million people), the so-called VAL databases (the Stockholm Region Healthcare Data Warehouse) ^27^. The VAL databases are a regional collection of administrative healthcare data, which, in part (specialist care), are regularly delivered to the national healthcare database at the National Patient Register ^25^. Since 2007, they have had nearly complete coverage of primary care and specialist care.

The study population consisted of all adult subjects ($\ge$ 18 years on February 28, 2018), diagnosed with hypertension with code I10.0 according to the International Classification of.

Diseases (ICD-10) during the period January 1, 2015–February 28, 2018, in Region Stockholm. Subjects with recorded diagnoses of diabetes mellitus type 1 and 2 (ICD–10 E10 and E11) and cardio– and cerebrovascular diseases (ICD–10 I11–I15, I20–I25, and I60–I69) during this period were excluded, as they were regarded as different patient groups. Patients who died or moved out of the region during this inclusion period were excluded from the study. This closed cohort, where no new patients were included during the study follow-up period, was followed during two distinct but continuous periods: pre-pandemic (from March 1, 2018, to February 29, 2020) and pandemic (from March 1, 2020, to February 28, 2022). In Sweden, most pandemic measures were issued as recommendations rather than legally binding restrictions, such as advice on remote work and temporary distance education for upper secondary schools and universities. Unlike many other countries, Sweden did not impose a formal lockdown; instead, authorities emphasized strict hand hygiene and physical distancing from the outset. At certain periods, however, mandatory restrictions were implemented, for example, limiting public gatherings such as funerals and theater events ^28,29^. Because these recommendations were not uniformly enforced and adherence may have varied, it was not possible to include them as formal exposure variables in our analyses.

### Outcomes and covariates

The outcome variables included alcohol-related disorders (see below) recorded in primary care or specialist inpatient care (including emergency care and hospitalizations), and deaths. In addition, other alcohol-associated conditions (see below), and also COVID-19, various infections, as well as causes of death known to be associated with alcohol drinking in clinical practice were selected ^12–15^.

The list of all diagnoses (ICD-10) included in this study is presented in Supplementary Table 1. In summary, alcohol-related disorders included: harmful use of alcohol (F10.1), alcohol dependency (F10.2), alcohol withdrawal-related diagnoses (F10.3–F10.5), and chronic alcohol usage related diagnoses (E51.2, G31.2, G62.1, G72.1, I42.6, I85.0, I85.9, K.85.2, and K86.0). Additionally, diagnoses related to intoxication due to non-alcohol substances (F11-F19) were included. The terminology of” alcohol-related disorders” has been used elsewhere ^30^.

Other alcohol-associated conditions included: cardiovascular disease such as ischemic heart diseases (I20–25), cerebrovascular diseases (I60–69), depression and anxiety (F32, F33, and F41), and injury- and accident-related diagnoses (S00–99 and T00–78). We also included COVID-19 (U07) and selected infectious diseases (A00–A09. A39–41, A87, B00–B34. B35–B49, G00–03, H60, H66, J00–01, J03, J06, J10, J12–J39, J20, N10, N12, N30, A46, and L03).

Finally, we studied death due to alcohol-related disorders (ICD-10 F10); cardio- and cerebrovascular diseases (I11–15, I20–25, and I60–I69), injury and accidents (S00–99, T00–T78), COVID-19 (U07), selected infectious diseases (A39–A41, A46, A87, G00–G03, J12–J39, L03, N10), deaths related to suicide (X60-X84), and deaths due to external causes of undetermined intent, such as poisoning, drowning (Y10-Y34).

Demographic information included age at the beginning of the pandemic (19–44, 45–64, 65–84, 85 and older), sex (male, female), education level ($\le$ 9, 10–12, > 12 years), employment status (employed, unemployed/retired), family status (living alone, living with others)**,** and country of birth (Sweden, Finland, other European Union countries (so-called EU28), US, Canada, and other countries).

### Ethical approval

This study is register-based and used pseudonymized data from the Swedish National Patient Register and primary care data from the administrative health data register of Region Stockholm, including Region Stockholm’s VAL databases, within the SCIFI-PEARL project framework. No participants were contacted, and no tissue samples were collected. The Swedish Ethical Review Authority approved the study (Dnr 2020–01800, with amendments) and waived the requirement for individual informed consent in accordance with GDPR. All analyses were performed on de-identified (pseudonymized) data obtained from the register owners and held by the SCIFI-PEARL project team; the research team had no access to directly identifiable information.

This study has been prepared in accordance with the STROBE guidelines.

### Statistical methods

The analysis of this study was conducted using descriptive time series analysis. The study population consisted of a closed cohort of participants with hypertension. Outcome diagnoses were identified as the first occurrence of each event within a 3-month interval, meaning that at least one diagnosis (at least one ICD-10 code) of that type during the period was counted for each patient, regardless of prior diagnoses. The quarterly period prevalence was estimated per 1,000 female or male participants with hypertension. For new diagnoses, such as COVID-19 or injury and accident, and events that occur only once, such as death, these were referred to as quarterly cumulative incidence. Due to a few events for some outcomes, the quarterly cumulative incidence of death due to suicide or poisoning, or drowning was estimated for 100,000 patients. All analyses were conducted using SAS 9.4 (SAS Institute, Cary, North Carolina, USA), with statistical significance assessed at a level of 5%.

## Results

### Characteristics of the study participants

A total of 168,963 adult participants from Region Stockholm, Sweden, were diagnosed with hypertension between January 1, 2015, and February 28, 2018, and included in this study (Table 1). Of these, 57% were female participants. The majority of participants were aged 65 to 84 years, with most being either unemployed or retired.

**Table 1:** Characteristics of study participants with hypertension (January 1, 2015–February 28, 2018) in Region Stockholm, Sweden.

Variables	N = 168 963	%
Sex		
Male	72 766	43.1
Female	96 197	56.9
Age at the pandemic (in years)		
19–44	6 435	3.8
45–64	54 507	32.3
65–84	89 695	53.1
≥ 85	18 326	10.9
Education level (in years)		
$\le$ 9	32 874	19.7
10–12	71 535	42.9
> 12	62 325	37.4
Missing	2 229	
Employment status		
Employed	68 092	40.3
Unemployed/retired	100 871	59.7
Family status		
Living alone	70 776	41.9
Living with others	98 187	58.1
Place of birth		
Sweden	129 656	76.7
Finland	10 601	6.3
EU28, US, Canada	7 649	4.5
Other countries	21 057	12.5

EU28- European Union countries other than Sweden and Finland.

### The trends of alcohol-related disorders and deaths

**Fig. 1:**
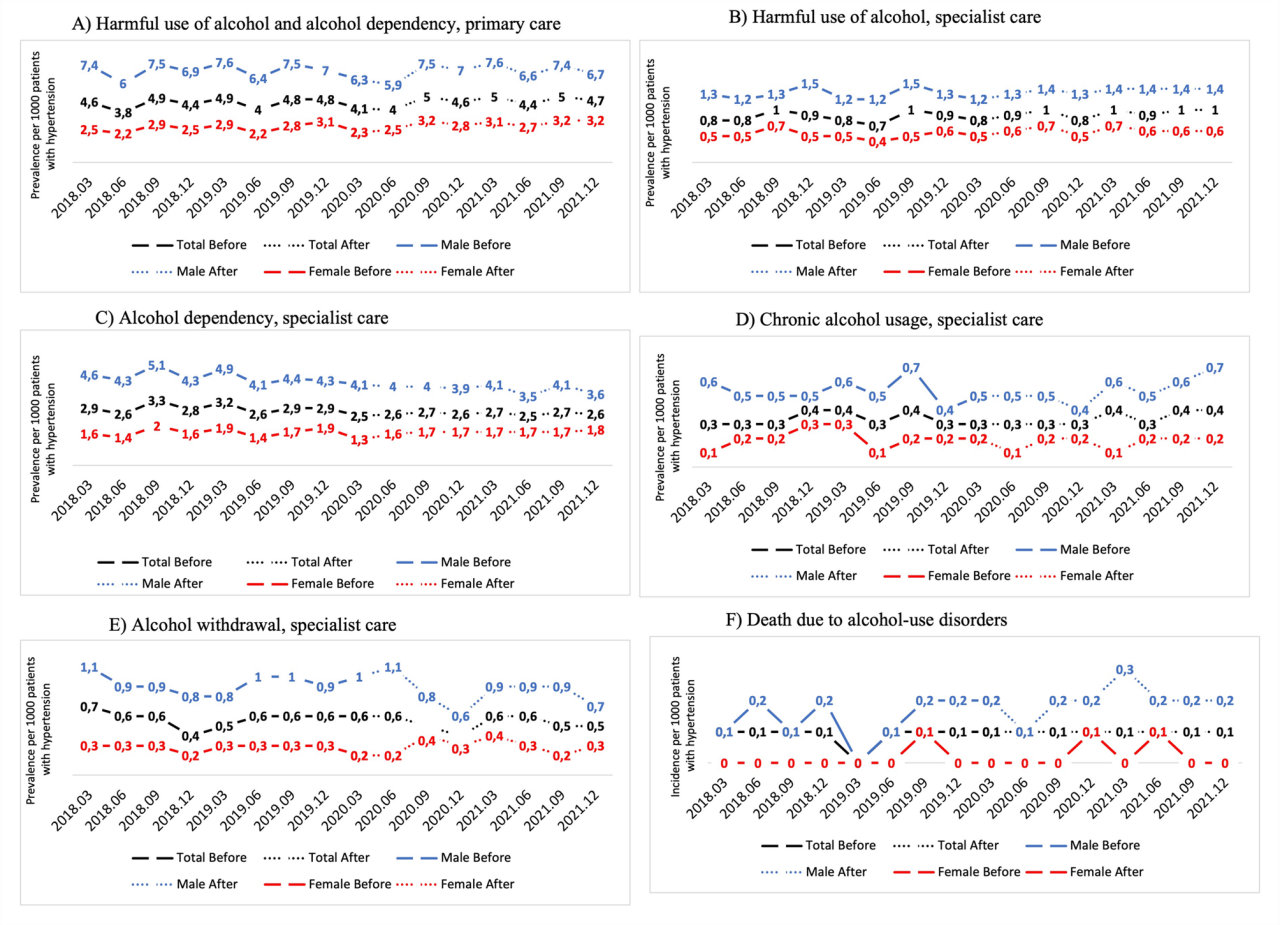
The quarterly period prevalence among study participants in Region Stockholm, Sweden, with hypertension (January 1, 2015–February 28, 2018) of a range of alcohol-related disorders per 1000 females and males during the COVID-19 pandemic (after), compared to the pre-pandemic period (before). (**A**) harmful use of alcohol and alcohol dependency (ICD-10 codes F10.1 and F10.2) in primary care; (**B**) harmful use of alcohol (F10.1) in specialist care; (**C**) alcohol dependency (F10.2) in specialist care; (**D**) alcohol withdrawal related disorders (F10.3–F10.5) in specialist care; (**E**) chronic alcohol usage related disorders (E51.2, G31.2, G62.1, G72.1, I42.6, I85.0, I85.9, K85.2, and K86.0) in specialist care; and (**F**) the quarterly cumulative incidence of deaths due to alcohol-use related disorders (F10).

The quarterly period prevalence of diagnoses related to harmful alcohol use and dependency decreased in primary care during the first two quarters of the pandemic to 2.3–2.5 per 1000 females and 6.3–5.9 per 1000 males, compared to 2.8–3.1 per 1000 females and 7.0–7.5 per 1000 males in the comparable pre-pandemic period. It then progressively returned to pre-pandemic levels in males, while showing an increasing trend in females (3.2 per 1000 females) during the last two quarters of 2021 (Fig. 1A).

The quarterly period prevalence of diagnoses related to harmful alcohol use and dependency in specialist care decreased during the first quarter of the pandemic to 1.2 per 1000 males and 0.5 per 1000 females, compared with 1.3 per 1000 males and 0.6 per 1000 females in the corresponding pre-pandemic period. Thereafter, rates rose, returning to pre-pandemic levels (Fig. 1B).

The periodic prevalence of the diagnosis of alcohol dependency registered in specialist care dropped during the pandemic period for males to 3.5–4.1 per 1000 males compared to 4.1–5.1 per 1000 males pre-pandemic, and females to 1.3–1.8 per 1000 females compared to 1.4–2.0 per 1000 females pre-pandemic (Fig. 1C).

The diagnosis of chronic alcohol usage remained unchanged in females during the pandemic (0.1–0.2 per 1000 females), whereas it dropped in the beginning of the pandemic in males to 0.4 per 100 males compared to 0.7 per 1000 males pre-pandemic and then returned to pre-pandemic levels by the end of the study period (Fig. 1D).

For the diagnosis of alcohol withdrawal, there was an initial decrease, yet after the first two quarters, we observed a peak to higher levels than pre-pandemic in females (0.4 per 1000 females compared to 0.2–0.3 per 1000 females pre-pandemic), which then fell back to pre-pandemic levels by the end of 2021. In contrast, in male participants, there was a slight increase at the beginning of the pandemic to 1.1 per 1000 males compared to 0.9–1.0 per 1000 males pre-pandemic, followed by a negative peak at the end of 2020 (0.6 per 1000 males) and then returned to 0.9 per 1000 males by the end of the pandemic study period (Fig. 1E).

The quarterly cumulative incidence of deaths due to alcohol-related disorders remained the same as before the pandemic (0–0.1 per 1000 females and 0.1–0.2 per 1000 males) (Fig. 1F), except for a slight peak in males at the beginning of 2021 (0.3 per 1000 males). Notably, the rates were higher in males than in female participants in both periods.

Additionally, the quarterly period prevalence of the diagnosis of dependency of non-alcoholic substances registered in secondary care did not change during the pandemic, being stable at 0.7–1.1 cases per 1000 males and 0.4–0.7 cases per 1000 females (Supplementary Fig. 1).

### The trends of diagnoses and deaths due to other alcohol-associated conditions

**Fig. 2:**
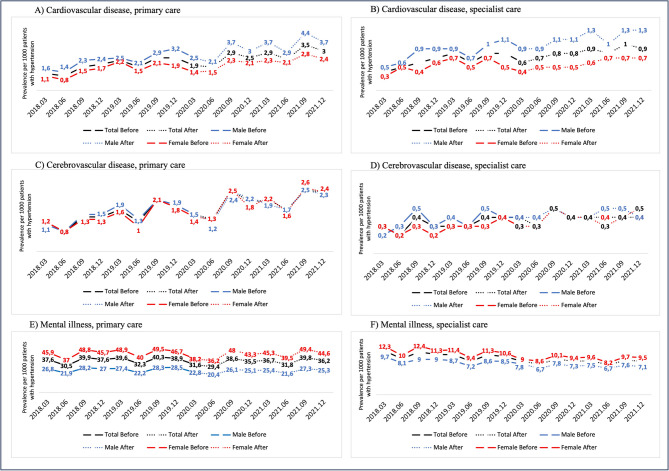
The quarterly period prevalence among study participants in Region Stockholm, Sweden, with hypertension (January 1, 2015–February 28, 2018) of some other alcohol-associated conditions, per 1000 females and males during the COVID-19 pandemic (after), compared to the pre-pandemic period (before). (**A**) cardiovascular disease (ICD-10 codes I20–25) in primary care; (**B**) cardiovascular disease (I20–25) in specialist care; (**C**) cerebrovascular diseases (I60-69) in primary care; (**D**) cerebrovascular diseases (I60–69) in specialist care; (**E**) mental illness (F32, F33, F41) in primary care; (**F**) mental illness (F32, F33, F41) in specialist care.

The quarterly period prevalence of cardiovascular diseases obtained in primary care decreased during the first six months of the pandemic (2.1–2.5 per 1000 males and 1.4–1.5 per 1000 females) compared to the pre-pandemic period (2.9–3.2 per 1000 males and 1.5–2.1 per 1000 females). Thereafter, by the end of 2021, both sexes showed an explicit increasing trend towards higher levels than before the outbreak, reaching 3.7–4.4 per 1000 males and 2.4–2.8 per 1000 females (Fig. 2A).

Similar trends were observed for cerebrovascular diseases obtained in primary care (Fig. 2C). The periodic prevalence was 1.4 per 1000 females and 1.5 per 1000 males at the beginning of the pandemic, compared to 1.8–2.1 per 1000 females and 1.9–2.1 per 1000 males just before the pandemic (Fig. 2C). By the end of the study period, it had increased to 2.6–2.4 per 1000 in both sexes.

The periodic prevalence of cardiovascular disease in specialist care initially dropped during the pandemic to 0.4 per 100 females and 0.9 per 1000 males, compared to 0.5–0.7 per females and 1.0–1.1 per 1000 males pre-pandemic. However, it later rose to clearly higher levels than pre-pandemic among male participants, reaching 1.3 per 1000 males by the end of the study period, while remaining similar to pre-pandemic levels for females (Fig. 2B).

The periodic prevalence of diagnoses of cerebrovascular disease in specialist care did not change during the pandemic compared to the pre-pandemic period, remaining at 0.3–0.5 per 1000 subjects in both sexes (Fig. 2D).

The quarterly cumulative incidence of deaths due to cardio- and cerebrovascular diseases remained on the same level, around 0.1–0.2 cases per 1000 subjects in both sexes (Supplementary Fig. 2).

The quarterly period prevalence of mental illness, including depression and anxiety diagnoses, obtained in all healthcare settings, clearly decreased at the beginning of the pandemic in both sexes. By the end of the pandemic study period, the rate of these diagnoses was lower than pre-pandemic levels, especially in specialist care (Fig. 2E–F). Specifically, the rates were 9.5 per 1000 females and 7.1 per 1000 males at the end of 2021, compared to 9.4–12.4 per 1000 females and 7.2–9.7 per 1000 males during the pre-pandemic study period (Fig. 2F).

### The trends of COVID-19 and other selected infectious diseases and related deaths

The quarterly cumulative incidence of COVID-19 diagnoses in primary care was higher in female than male participants during 2020–2021 (Supplementary Fig. 3). However, the need for emergency care and hospitalization due to COVID-19 was more frequent in males than females, particularly during the first year of the pandemic, 2020 (Supplementary Fig. 4). The deaths due to COVID-19 were highest in the first months of the pandemic, affecting both sexes (3.1 per 1000 females and 2.8 per 1000 males) (Supplementary Fig. 5).

The quarterly cumulative incidence of selected infectious diseases, apart from COVID-19, registered in different care settings decreased during the pandemic compared to the time before the outbreak, in all participants (Supplementary Figs. 6 and 7). Then, the death rate was highest in the first quarter of the pandemic in both sexes, being 1.1 cases per 1000 subjects (Supplementary Fig. 8).

### The quarterly cumulative incidence of diagnoses and deaths related to injury and accident

The quarterly cumulative incidence of diagnoses related to injury and accident obtained in specialist care decreased in the first six months of the pandemic, but after that, an increased trend was seen in all participants (at the end of 2021 it was 8.4 cases per 1000 females and 6.7 cases per 1000 males) compared to the period before the outbreak (in 2019–2020 it was 5.5–7.4 cases per 1000 in females and 4.2–5.4 cases in males; Supplementary Fig. 9). The deaths due to injury and accident remained unchanged during the pandemic (Supplementary Fig. 10).

The suicide diagnosis fluctuated over time and remained unchanged during the pandemic in comparison to the period before the outbreak (Supplementary Fig. 11). Furthermore, deaths due to external causes of undetermined intent (such as poisoning, drowning, etc.) increased dramatically in the first quarter of the pandemic in all participants. Later in the pandemic, the occurrence of these diagnoses returned to the same levels as before the outbreak in female participants; however, it changed its character in males, and remained at a level higher than the pre-pandemic in male participants (Supplementary Fig. 12).

## Discussion

From a public health perspective, and considering potential prevention strategies, this study focused on a vulnerable group—patients with hypertension. Our main finding revealed no profound shift overall in alcohol-related disorders or deaths from the causes studied during the pandemic compared to the pre-pandemic period. However, some changes were apparent. In female participants, the quarterly period prevalence of alcohol-related disorders in primary care showed an upward trend. In contrast, the trend of alcohol dependency in specialist care appeared to decline, particularly in males. Both during the pandemic and the pre-pandemic period, the general rates of all alcohol-related disorders and alcohol-related deaths were higher in males than in females. In addition, among other alcohol-associated conditions, cardiovascular disease exhibited an increasing trend in both sexes in primary care and in males in specialist care. Mental illness showed decreasing trends in both sexes, particularly in specialist care.

To our knowledge, this is the first study to examine pandemic-related trends in alcohol-related disorders among participants with hypertension, a large and heterogeneous group particularly vulnerable to alcohol. Unlike a study conducted in the general U.S. population, which covered a similar pandemic period as ours ^31^, we did not find any increase, but rather a decrease in males, of any of the alcohol-related disorders in specialist care. The US study, which employed a longitudinal interrupted time series analysis, reported significantly higher-than-expected rates of alcohol-related complications during the pandemic for both males and females, with significant increases particularly among females aged 40 to 64 ^31^. In alignment with these results, a meta-analysis on alcohol intake showed an increase of harmful drinking in the US, but no increase has been reported in Europe ^20,32,33^.

In our study, we observed an upward trend in alcohol-related disorder diagnoses in primary care among females. This increase may not be directly related to the pandemic, but rather reflects a broader pattern in Sweden. Between 2011 and 2022, 15.6% of the Swedish general population reported hazardous alcohol use. While the prevalence has declined in men, it has remained unchanged in women ^34^. Additionally, hazardous alcohol use is more frequent in individuals with hypertension compared to the general population ^7^. In Sweden, approximately 27% of adults have hypertension ^35^, with most cases managed in primary care ^36^. Primary care plays a vital role in addressing lifestyle factors and promoting healthier habits, such as reducing alcohol consumption ^37^; however, this was disrupted during the pandemic and could contribute to shifts in alcohol-related disorders.

We found that death due to alcohol-related disorders did not change during the pandemic in either sex. This finding aligns with data from two other Scandinavian countries, Denmark and Finland, included in a meta-analysis ^38^. In contrast, alcohol-attributed deaths increased in the US ^39^ and some European countries, particularly the Baltic countries, Bulgaria, and Spain ^38^. It is important to consider that some alcohol-related disorders may have been categorized as COVID-19 cases on the death certificate during the pandemic when these conditions co-occurred.

Our study found that most other alcohol-associated conditions decreased across healthcare settings during the first six months of the pandemic. However, as the pandemic progressed, the periodic prevalence of many diagnoses returned to pre-pandemic levels, with one notable exception: cardiovascular diagnoses, which increased in both sexes in primary care and in males in specialist care. This trend was observed across all healthcare settings and may be attributed to delayed care during the pandemic ^24,40^, as healthcare systems focused on controlling COVID-19, reducing routine screenings and diagnostics ^41^. In Sweden, the overall rate of physical healthcare visits decreased during the early phase of the pandemic, as the Public Health Agency recommended in March 2020 that all non-essential care should be postponed ^42^. This likely affected the management of patients with alcohol-related disorders and chronic cardiovascular diseases, and may also have delayed the diagnosis of new cases.

Once healthcare services resumed normal operations, these common diagnoses increased as patients finally sought care ^43^, potentially leading to missed opportunities for preventive cardiovascular care in individuals with high risk. The long-term health consequences of decreased prevention during the pandemic remain unknown.

In line with another Swedish study in the general population ^44^, we observed that hospitalization rates for cerebrovascular disease remained stable among subjects with hypertension during the pandemic, comparable to the pre-pandemic period. In terms of mortality from cardio- and cerebrovascular diseases, our study found no significant change in deaths during the pandemic compared to the pre-outbreak period. These findings align with results from the Swedish national registry study in the general population, which reported no significant change in death rates due to cerebrovascular and cardiovascular diseases, with a slight trend towards a decrease ^45^.

Our study found that the periodic prevalence of the most common mental illnesses, depression and anxiety, decreased in both males and females with hypertension early in the pandemic and stayed lower than before by the end of the pandemic study period, especially in specialist care. Patients with emerging mental health or substance-related conditions may have postponed seeking care, resulting in delayed identification of these diagnoses. We showed that, similar to pre-pandemic trends, the occurrence of diagnoses of depression and anxiety continued to be higher in females than in males. The association between alcohol-related disorders and depression and anxiety is well-documented ^46^. Although fears, financial concerns, and isolation during the pandemic were thought to harm mental health ^47^, a survey study across six countries, including Sweden, found no overall change in depression and anxiety symptom rates, apart from higher symptom rates in those with self-reported prior diagnoses of depression/anxiety ^48^. Similarly, a United Kingdom (UK) study reported fewer newly diagnosed psychiatric diagnoses during COVID-19 ^49^. As an explanation, it has been suggested that individuals with milder mental health issues may have sought alternative coping mechanisms outside of medical intervention. Additionally, access to healthcare was limited due to the prioritization of COVID-19 ^24,40^.

Our study also showed that female participants were more frequently diagnosed with COVID-19 in primary care throughout the pandemic, while male participants were more commonly hospitalized due to the infection. This finding aligns with previous meta-analyses indicating that males accounted for a higher proportion of severe and critical cases ^50^. Mortality rates in our study varied between hypertensive females and males, depending on the study period. This pattern contrast with the general population, where both international and Swedish statistics reported that male mortality rates were up to twice as high as those for females during the pandemic ^51^ This difference might be explained by the fact that hypertensive patients often have additional chronic conditions compared to the general population ^52^, a pattern also observed in our study cohort.

We further demonstrated that the cumulative incidence of injuries and accidents requiring emergency care or hospitalization increased in the latter part of the pandemic for both sexes, compared to the pre-pandemic period. Previous research suggested that as people spent more time at home, there was an increase in domestic injuries and accidents, especially falls and ingestions, while vehicle-related accidents declined ^53,54^. Despite this increase in non-fatal incidents, we observed that deaths due to injury and accidents remained unchanged during the pandemic compared to pre-pandemic levels. In Sweden, strict lockdowns were not implemented, and mobility restrictions were less pronounced than in many other countries. Our findings highlight the complex and context-specific impact of the pandemic on accident and injury epidemiology, and underline the importance of considering national strategies and healthcare-seeking behaviors when interpreting the results.

Hazardous alcohol use has also been strongly linked to suicides—evidenced by a pre-pandemic meta-analysis showing a 94% increase in the risk of suicide related to alcohol use ^13^. The COVID-19 pandemic represented a unique societal event that may have influenced these outcomes in complex ways. Therefore, given the established association between alcohol use and an elevated risk of suicide, we also analyzed suicide-related deaths. Our findings revealed that, as in the pre-pandemic period, suicide rates fluctuated and remained consistently higher in males than in females. Despite concerns that social isolation and economic stress would increase suicide rates, both our findings and others in the general population indicate that suicide incidence remained stable during the pandemic ^55^. This suggests that while alcohol use is a well-established risk factor for suicide, the pandemic did not substantially alter overall trends in suicide mortality, at least in the short term.

The clinical relevance of our findings lies in their ability to inform tailored prevention and management strategies for hypertensive patients, a population already at heightened risk for adverse cardiovascular outcomes ^5^. The observed sex-specific differences—such as the rise in alcohol-related disorders in females within primary care and the increase in cardiovascular diagnoses among males in specialist care—highlight the need for gender-sensitive approaches in clinical practice. These insights reinforce the critical role of primary care in addressing lifestyle factors, such as alcohol consumption, which remains a modifiable risk factor for cardiovascular disease ^7^. Elevated blood pressure remains the leading preventable cause of cardiovascular mortality globally ^2^. Moreover, the study underscores the broader impact of the COVID-19 pandemic on healthcare delivery, emphasizing how disruptions in routine care may have contributed to delayed diagnoses and potential missed opportunities for preventive interventions ^24,40^. Long-term cardiovascular monitoring of hypertensive patients is essential, as the pandemic may have compounded risks through increased stress and lifestyle changes such as reduced physical activity and altered nutrition.

Furthermore, COVID-19 itself may result in long-term health effects, including hypertension, other cardiovascular disorders ^56^ and post-COVID-19 condition ^57^. These findings stress the importance of maintaining uninterrupted care for chronic conditions during public health crises and highlight the need for further research to fully understand the long-term health consequences in vulnerable populations. Further research—including studies covering the post-pandemic period and incorporating risk factors other than alcohol—is necessary to fully understand the impact of the COVID-19 pandemic and its countermeasures on other alcohol-associated conditions, including cardiovascular diseases, in the population.

The strengths of our study lie in the use of comprehensive, large population-based data on patients with hypertension from both urban and rural areas within Region Stockholm, Sweden. This approach ensures robust representation across different age groups and sexes, enabling a detailed examination of trends over time in females and males. The repeated quarterly data from the Swedish national registers also allow for unique insights into the various phases of the pandemic. Our analysis also captures diagnoses originating from both primary and specialist care settings.

This study has several limitations, primarily due to its descriptive and exploratory character, which restricts causal interpretations of the observed trends. Another limitation is that our data did not capture alcohol-related disorders for patients who did not seek medical care. While surveys can provide such information, our findings align well with survey-based study findings. Furthermore, we did not account for different virus variants across the quarterly periods. We did not account for possible changes in attainment of blood pressure targets, a phenomenon observed in the Stockholm Region during the pandemic ^58^. We did not adjust for antihypertensive treatment as a variable in our analyses. Although treatment status could be represented as a binary factor, accounting for it properly would require a different study design to disentangle treatment effects from underlying disease severity and health-related behaviors, which is a different scientific question. Finally, our register-based study did not account for other lifestyle factors that may have changed during the pandemic, such as decreased physical activity, altered nutritional behaviors, or an increase in BMI, which could all contribute to different health trajectories in hypertensive individuals.

## Conclusions

In conclusion, this is the first exploratory study to examine pandemic changes in alcohol-related disorders and other alcohol-associated conditions, as well as deaths, in all healthcare settings in a heterogeneous and vulnerable population of patients with hypertension. Consistent with pre-pandemic trends, we showed that male participants had a higher prevalence than female adults of all alcohol-related disorders. Noteworthy results were that among females, the periodic prevalence of alcohol-related disorders in primary care tended to increase, and alcohol dependency in specialist care decreased by the end of the pandemic study period in both sexes, particularly males. Of other alcohol-associated conditions, cardiovascular disease diagnoses increased in both sexes in primary care and in male participants in specialist care, whereas mental illness decreased in both sexes, particularly in specialist care. However, due to the snapshot design of this study, it is challenging to draw definitive conclusions, as pandemic-related health impacts may not become apparent until many years later.

## Supplementary Information

Supplementary Information.

## Data Availability

Data is not available from the Authors, but we welcome collaborations where we analyze the data. Our data may not be shared outside of Karolinska Institutet for ethical reasons realuted via GDPR and Swedish laws on health-care registers. The data from this study can be obtained for research purposes by qualified researchers after obtaining ethical approval from Region Stockholm at [halsodata.rst@regionstockholm.se]([mailto:halsodata.rst@regionstockholm.se]) .

## References

[1] Collaboration, N. C. D. R. F. Worldwide trends in hypertension prevalence and progress in treatment and control from 1990 to 2019: A pooled analysis of 1201 population-representative studies with 104 million participants. *Lancet* **398**(10304), 957–980 (2021). 34450083 10.1016/S0140-6736(21)01330-1 PMC8446938

[2] Collaborators, G. B. D. R. F. Global burden of 87 risk factors in 204 countries and territories, 1990–2019: A systematic analysis for the Global Burden of Disease Study 2019. *Lancet* **396**(10258), 1223–1249 (2020). 33069327 10.1016/S0140-6736(20)30752-2 PMC7566194

[3] WHO, *Global Report on Hypertension, Last Seen 2024–11–30* (2023).

[4] Kario, K. et al. The WHO Global report 2023 on hypertension warning the emerging hypertension burden in globe and its treatment strategy. *Hypertens. Res.* **47**(5), 1099–1102 (2024). 38443614 10.1038/s41440-024-01622-w

[5] Williams, B. et al. 2018 ESC/ESH guidelines for the management of arterial hypertension: The task force for the management of arterial hypertension of the European society of cardiology and the european society of hypertension: The task force for the management of arterial hypertension of the European society of cardiology and the European society of hypertension. *J. Hypertens.* **36**(10), 1953–2041 (2018). 30234752 10.1097/HJH.0000000000001940

[6] Lim, S. S. et al. A comparative risk assessment of burden of disease and injury attributable to 67 risk factors and risk factor clusters in 21 regions, 1990–2010: A systematic analysis for the Global Burden of Disease Study 2010. *Lancet* **380**(9859), 2224–2260 (2012). 23245609 10.1016/S0140-6736(12)61766-8 PMC4156511

[7] Sven Andreasson, F. D., Timothy, N. Aalcohol and blood pressure, in *Alcohol and Society 2023*, I.a.S. Research, Editor (2023): https://movendi.ngo/wp-content/uploads/2023/03/IOGT-11400-Rapport_2023_EN-1.pdf

[8] Organization, W. H., *Global Status Report on Alcohol and Health and Treatment of Substance Use Disorders* (2024). https://www.who.int/publications/i/item/9789240096745

[9] Rehm, J. et al. Alcohol as a risk factor for global burden of disease. *Eur. Addict. Res.* **9**(4), 157–164 (2003). 12970584 10.1159/000072222

[10] Puddey, I. B. & Beilin, L. J. Alcohol is bad for blood pressure. *Clin. Exp. Pharmacol. Physiol.* **33**(9), 847–852 (2006). 16922819 10.1111/j.1440-1681.2006.04452.x

[11] Puddey, I. B. et al. Alcohol and hypertension-new insights and lingering controversies. *Curr. Hypertens. Rep.* **21**(10), 79 (2019). 31494743 10.1007/s11906-019-0984-1

[12] Organization., W.H., *Highlights Glaring Gaps in the Regulation of Alcohol Marketing Across Borders* (2023). https://www.who.int/news/item/10-05-2022-who-highlights-glaring-gaps-in-regulation-of-alcohol-marketing-across-borders

[13] Isaacs, J. Y. et al. Alcohol use and death by suicide: A meta-analysis of 33 studies. *Suicide Life Threat Behav.* **52**(4), 600–614 (2022). 35181905 10.1111/sltb.12846

[14] Strasiotto, L. et al. The role of alcohol and drug intoxication in fatal drowning and other deaths that occur on the Australian coast. *J. Saf. Res.* **82**, 207–220 (2022). 10.1016/j.jsr.2022.05.012 36031248

[15] Patra, J. et al. Alcohol consumption and the risk of morbidity and mortality for different stroke types–a systematic review and meta-analysis. *BMC Public Health* **10**, 258 (2010). 20482788 10.1186/1471-2458-10-258 PMC2888740

[16] Organization, W. H. *WHO. Coronavirus Disease (COVID-19)* (World Health Organization, 2020).

[17] Shield, K. et al. National, regional, and global burdens of disease from 2000 to 2016 attributable to alcohol use: a comparative risk assessment study. *Lancet Public Health* **5**(1), e51–e61 (2020). 31910980 10.1016/S2468-2667(19)30231-2

[18] Roberts, A. et al. Alcohol and other substance use during the COVID-19 pandemic: A systematic review. *Drug Alcohol. Depend.* **229**(Pt A), 109150 (2021). 34749198 10.1016/j.drugalcdep.2021.109150 PMC8559994

[19] Nindenshuti, P. M. & Caire-Juvera, G. Changes in diet, physical activity, alcohol consumption, and tobacco use in adults during the COVID-19 pandemic: A systematic review. *Inquiry* **60**, 469580231175780 (2023). 37219073 10.1177/00469580231175780 PMC10208950

[20] Kilian, C. et al. Changes in alcohol use during the COVID-19 pandemic in Europe: A meta-analysis of observational studies. *Drug Alcohol. Rev.* **41**(4), 918–931 (2022). 35187739 10.1111/dar.13446 PMC9111882

[21] Rossow, I. et al. Changes in alcohol consumption during the COVID-19 pandemic-small change in total consumption, but increase in proportion of heavy drinkers. *Int J. Environ. Res. Public Health* **18**(8), 4231 (2021). 33923567 10.3390/ijerph18084231 PMC8073387

[22] Sohal, A. et al. Impact of COVID-19 pandemic on alcohol-related hepatitis admissions: Analysis of nationwide data 2016–2020. *Am. J. Med. Sci.* **366**(3), 209–218 (2023). 37315782 10.1016/j.amjms.2023.06.002 PMC10259164

[23] Marlowe, N., Lam, D. & Liangpunsakul, S. Epidemic within a pandemic: Alcohol-associated hepatitis and COVID-19. *Alcohol. Clin. Exp. Res. (Hoboken)* **47**(10), 1883–1889 (2023). 37553753 10.1111/acer.15162

[24] Gotanda, H. et al. Changes in blood pressure outcomes among hypertensive individuals during the COVID-19 pandemic: A time series analysis in three US healthcare organizations. *Hypertension* **79**(12), 2733–2742 (2022). 36317526 10.1161/HYPERTENSIONAHA.122.19861 PMC9640259

[25] Nyberg, F. et al. Swedish covid-19 investigation for future insights - A population epidemiology approach using register linkage (SCIFI-PEARL). *Clin. Epidemiol.* **13**, 649–659 (2021). 34354377 10.2147/CLEP.S312742 PMC8331198

[26] Ludvigsson, J. F. et al. External review and validation of the Swedish national inpatient register. *BMC Public Health* **11**, 450 (2011). 21658213 10.1186/1471-2458-11-450 PMC3142234

[27] Forslund, T. et al. Risk scoring and thromboprophylactic treatment of patients with atrial fibrillation with and without access to primary healthcare data: Experience from the Stockholm health care system. *Int. J. Cardiol.* **170**(2), 208–214 (2013). 24239153 10.1016/j.ijcard.2013.10.063

[28] Hallberg, A. et al. Epidemiological outcomes and policy implementation in the Nordic countries during the COVID-19 pandemic. *Arch Public Health* **83**(1), 46 (2025). 39980066 10.1186/s13690-025-01531-5 PMC11844186

[29] Ludvigsson, J. F. How Sweden approached the COVID-19 pandemic: Summary and commentary on the National Commission Inquiry. *Acta Paediatr.* **112**(1), 19–33 (2023). 36065136 10.1111/apa.16535 PMC9538368

[30] Bergman, D. et al. Incidence of ICD-based diagnoses of alcohol-related disorders and diseases from Swedish nationwide registers and suggestions for coding. *Clin. Epidemiol.* **12**, 1433–1442 (2020). 33408530 10.2147/CLEP.S285936 PMC7781026

[31] Shuey, B. et al. High-acuity alcohol-related complications during the COVID-19 pandemic. *JAMA Health Forum* **5**(4), e240501 (2024). 38607643 10.1001/jamahealthforum.2024.0501 PMC11065164

[32] Sohi, I. et al. Changes in alcohol use during the COVID-19 pandemic and previous pandemics: A systematic review. *Alcohol Clin. Exp. Res.* **46**(4), 498–513 (2022). 35412673 10.1111/acer.14792 PMC9111333

[33] Granstrom, F. et al. Impact of the pandemic on leisure physical activity and alcohol consumption. *BMC Public Health* **24**(1), 1589 (2024). 38872148 10.1186/s12889-024-19100-w PMC11177532

[34] (folkhälsomyndigheter), P.h.a.o.s. *Alcohol Use*. 2022 2024-09-28].

[35] Kahan, T. D. H., deFaire U. et al. *Moderately Elevated Blood Pressure. A Systematic Literature Review. Stockholm: The Swedish Council on Technology Assessment in Health Care 2004–Update 2007. Report 170/1U:1–195. Report 170/1U:1–195*.

[36] Carlsson, A. C. et al. High prevalence of diagnosis of diabetes, depression, anxiety, hypertension, asthma and COPD in the total population of Stockholm, Sweden - a challenge for public health. *BMC Public Health* **13**, 670 (2013). 23866784 10.1186/1471-2458-13-670 PMC3724714

[37] Brobeck, E. et al. Health promotion practice and its implementation in Swedish health care. *Int Nurs Rev* **60**(3), 374–380 (2013). 23961800 10.1111/inr.12041

[38] Kilian, C. et al. Changes in alcohol-specific mortality during the COVID-19 pandemic in 14 European countries. *Sucht* **69**(6), 285–293 (2023). 39183774 10.1024/0939-5911/a000841 PMC11343567

[39] White, A. M. et al. Alcohol-related deaths during the COVID-19 pandemic. *JAMA* **327**(17), 1704–1706 (2022). 35302593 10.1001/jama.2022.4308 PMC8933830

[40] Trimarco, V. et al. Incidence of new-onset hypertension before, during, and after the COVID-19 pandemic: A 7-year longitudinal cohort study in a large population. *BMC Med* **22**(1), 127 (2024). 38500180 10.1186/s12916-024-03328-9 PMC10949764

[41] Lim, J. et al. COVID-19’s impact on primary care and related mitigation strategies: A scoping review. *Eur. J. Gen. Pract.* **27**(1), 166–175 (2021). 34282695 10.1080/13814788.2021.1946681 PMC8293960

[42] Ludvigsson, J. F. The first eight months of Sweden’s COVID-19 strategy and the key actions and actors that were involved. *Acta Paediatr.* **109**(12), 2459–2471 (2020). 32951258 10.1111/apa.15582 PMC7537539

[43] Jia, R. et al. Mental health in the UK during the COVID-19 pandemic: Cross-sectional analyses from a community cohort study. *BMJ Open* **10**(9), e040620 (2020). 32933965 10.1136/bmjopen-2020-040620 PMC7493070

[44] Rydell, M. et al. Maintained acute stroke admission during the first wave COVID-19 pandemic in Sweden, a register-based study. *J. Stroke Cerebrovasc. Dis.* **31**(10), 106686 (2022). 35933763 10.1016/j.jstrokecerebrovasdis.2022.106686 PMC9325685

[45] Axenhus, M. et al. Changes in mortality trends amongst common diseases during the COVID-19 pandemic in Sweden. *Scand. J. Public Health* **50**(6), 748–755 (2022). 34933630 10.1177/14034948211064656 PMC9361422

[46] Grazioli, V. S. et al. Depressive symptoms, alcohol use and coping drinking motives: Examining various pathways to suicide attempts among young men. *J. Affect. Disord.* **232**, 243–251 (2018). 29499507 10.1016/j.jad.2018.02.028

[47] Kamble, S. et al. Influence of COVID-19 pandemic on psychological status: An elaborate review. *Cureus* **14**(10), e29820 (2022). 36337829 10.7759/cureus.29820 PMC9622468

[48] Gemes, K. et al. Symptoms of anxiety and depression during the COVID-19 pandemic in six European countries and Australia - Differences by prior mental disorders and migration status. *J. Affect. Disord.* **311**, 214–223 (2022). 35598751 10.1016/j.jad.2022.05.082 PMC9119165

[49] Williams, R. et al. Diagnosis of physical and mental health conditions in primary care during the COVID-19 pandemic: A retrospective cohort study. *Lancet Public Health* **5**(10), e543–e550 (2020). 32979305 10.1016/S2468-2667(20)30201-2 PMC7511209

[50] Galbadage, T. et al. Systematic review and meta-analysis of sex-specific COVID-19 clinical outcomes. *Front. Med. (Lausanne)* **7**, 348 (2020). 32671082 10.3389/fmed.2020.00348 PMC7331754

[51] Socialstyrelsen, *Statistics on COVID-19*. 2023.

[52] Lauder, L. et al. Hypertension management in patients with cardiovascular comorbidities. *Eur. Heart J.* **44**(23), 2066–2077 (2023). 36342266 10.1093/eurheartj/ehac395

[53] Gielen, A. C. et al. National survey of home injuries during the time of COVID-19: Who is at risk?. *INJ Epidemiol.* **7**(1), 63 (2020). 33176881 10.1186/s40621-020-00291-w PMC7656093

[54] Yasin, Y. J., Grivna, M. & Abu-Zidan, F. M. Global impact of COVID-19 pandemic on road traffic collisions. *World J. Emerg. Surg.* **16**(1), 51 (2021). 34583713 10.1186/s13017-021-00395-8 PMC8478263

[55] Yan, Y. et al. Suicide before and during the COVID-19 Pandemic: A systematic review with meta-analysis. *Int. J. Environ. Res. Public Health* **20**(4), 3346 (2023). 36834037 10.3390/ijerph20043346 PMC9960664

[56] Al-Aly, Z., Xie, Y. & Bowe, B. High-dimensional characterization of post-acute sequelae of COVID-19. *Nature* **594**(7862), 259–264 (2021). 33887749 10.1038/s41586-021-03553-9

[57] Kisiel, M. A. et al. Clustering analysis identified three long COVID phenotypes and their association with general health status and working ability. *J. Clin. Med.* **12**(11), 3617 (2023). 37297812 10.3390/jcm12113617 PMC10253616

[58] Kunskapsstyrning, N. S. F. *Primärvårdsrapport 2023 Regionalt Programområde Primärvård, Sjukvårdsregion Stockholm-Gotland* (2024).

