# Supplementary material for: Impact of the COVID-19 pandemic on trends in health conditions associated with alcohol among patients with hypertension in Sweden
Source: Sci Rep. 2025 Oct 1;15:34295. doi: 10.1038/s41598-025-21712-0 (PMC12488861; doi:10.1038/s41598-025-21712-0)
Supplement: Supplementary file 1 — Supplementary Information. [file 41598_2025_21712_MOESM1_ESM.docx]

Supplementary materials:

| Supplementary Table 1: Study outcome diagnoses (ICD-10), including alcohol-related disorders and other alcohol-associated conditions in study participants in Region Stockholm, Sweden, with hypertension (Jan 1, 2015 - Feb 28, 2018). Diagnoses were obtained from different care forms, including primary and specialist care. | | | |
| --- | --- | --- | --- |
| **Group of disorders** | **ICD-10** | **Diagnoses** | **Care level** |
| **Alcohol-related disorders** |  |  |  |
|  | F10.1 | Harmful use of alcohol | All care |
|  | F10.2 | Alcohol dependency | All care |
|  |  | Alcohol withdrawal related diagnoses |  |
|  | F10.3 | Alcohol withdrawal | Specialist care |
|  | F10.4 | Alcohol withdrawal state with delirium | Specialist care |
|  | F10.5 | Psychotic disorder due to alcohol | Specialist care |
|  |  | Chronic alcohol usage related diagnoses | Specialist care |
|  | E51.2 | Wernicke’s encephalopathy | Specialist care |
|  | G31.2 | Degeneration of nervous system due to alcohol | Specialist care |
|  | G62.1 | Alcoholic neuropathy | Specialist care |
|  | G72.1 | Alcoholic myopathy | Specialist care |
|  | I42.6 | Alcoholic cardiomyopathy | Specialist care |
|  | I85.0 | Oesophagal varices with bleeding | Specialist care |
|  | I85.9 | Oesophagal varices without bleeding | Specialist care |
|  | K85.2 | Alcohol-induced acute pancreatitis | Specialist care |
|  | K86.0 | Alcohol-induced chronic pancreatitis | Specialist care |
| **Other alcohol-associated conditions** |  |  |  |
| Cardiovascular disease | I20-25 | Ischemic heart diseases | All care |
| Cerebrovascular disease | I60-69 | Cerebrovascular diseases | All care |
| Mental illness | F32 | Depressive episodes, | All care |
|  | F33 | Major depressive disorders, recurrent | All care |
|  | F41 | Anxiety disorders | All care |
| Intoxication | F11-F19. | Dependency, various substances, not alcohol | Specialist care |
| Selected infectious diseases | A00-A09 | Infectious intestinal diseases | All care |
|  | B00-B34 | Viral infections | All care |
|  | B35-B49 | Mycoses, | All care |
|  | H60, H66, J00-01, J03, J06 | Acute upper respiratory tract infections | All care |
|  | J10 | Influenza | All care |
|  | J15, J18 | Bacterial pneumonia, Pneumonia | All care |
|  | J12-J39 | Pneumonia, bacterial and viral | Specialist care |
|  | J20 | Acute bronchitis, various | Specialist care |
|  | N10, N12 | Acute nephritis | All care |
|  | N30 | Cystitis | All care |
|  | A46, L03 | Cellulitis | All care |
|  | A39-A41 | Meningococcal infection, streptococcal and other bacterial septicemias | Specialist care |
|  | A87 | Viral meningitis | Specialist care |
|  | G00-G03 | Meningitis, bacterial, viral and fungal | Specialist care |
|  | U07 | COVID-19 | All care |
| Injuries and accidents | S00-99 | Injuries on body parts, specified | Specialist care |
|  | T00-78 | Effects of external injuries. | Specialist care |
| **Causes of death** |  |  | NA |
|  | U07 | COVID-19 | NA |
|  | F10 | Alcohol-related disorders | NA |
|  | I11-15, I20-25, I60-I69 | Cardio- and cerebrovascular diseases | NA |
|  | S00-99, T00-T78 | Accidents | NA |
|  | A39-A41, A46, A87, G00-G03, J12-J39, L03, N10 | Infections |  |
|  | X60-X84 | Intentional self-harm, suicide | NA |
|  | Y10-Y34 | Events of undetermined intent (such as poisoning, drowning, etc.) | NA |

Note: NA- not applicable.

Supplementary Figure 1: The quarterly period prevalence among study participants in Region Stockholm, Sweden, with hypertension (Jan 1, 2015 - Feb 28, 2018) of substance dependency except alcohol (ICD-10: F11-F19: Dependency, various substances) in specilits care, per 1000 females and males.

Supplementary Figure 2: The quarterly cumulative incidence among study participants in Region Stockholm, Sweden, with hypertension (Jan 1, 2015 - Feb 28, 2018) of death due to cardio- and cerebrovascular diseases (ICD-10: I11-15, I20-25, I60-I69), per 1000 females and males.

Supplementary Figure 3: The quarterly cumulative incidence among study participants in Region Stockholm, Sweden, with hypertension (Jan 1, 2015 - Feb 28, 2018) of COVID-19 (ICD-10: U07: COVID-19) in primary care, per 1000 females and males.

Supplementary Figure 4: The quarterly cumulative incidence among study participants in Region Stockholm, Sweden, with hypertension (Jan 1, 2015 - Feb 28, 2018) of COVID-19 (ICD-10: U07: COVID-19) in specialist care, per 1000 females and males.

Supplementary Figure 5: The quarterly cumulative incidence among study participants in Region Stockholm, Sweden, with hypertension (Jan 1, 2015 - Feb 28, 2018) of death due to COVID-19 (ICD-10: U07), per 1000 females and males.

Supplementary Figure 6: The quarterly cumulative incidence among study participants in Region Stockholm, Sweden, with hypertension (Jan 1, 2015 - Feb 28, 2018) of infectious diseases other than COVID-19 (ICD-10: A00-A09: Infectious intestinal diseases, B00-B34: Viral infections, var., B35-B49: Mycoses, H60, H66, J00-01, J03, J06: Acute upper respiratory tract infections, var., J10: Influenza, J12-J39: Pneumonia, bacterial and viral, N10, N12: Acute nephritis and N30: Cystitis, L03: Cellulitis and acute lymphangitis) in primary care, per 1000 females and males.

Supplementary Figure 7: The quarterly cumulative incidence among study participants in Region Stockholm, Sweden, with hypertension (Jan 1, 2015 - Feb 28, 2018) of infectious diseases (ICD-10: A00-A09, A39-41, A46, A87, B00-B34. B35-B49, G00-G03, H60, H66, J00, J03, J05, J10, J12-29, L03, N10, N12 and N30) in specialist care, per 1000 females and males.

Supplementary Figure 8: The quarterly cumulative incidence among study participants in Region Stockholm, Sweden, with hypertension (Jan 1, 2015 - Feb 28, 2018) of death due to selected infectious diseases (ICD-10: A39-A41, A46, A87, G00-G03, J12-J39, L03, N10), per 1000 females and males.

Supplementary Figure 9: The quarterly cumulative incidence among study participants in Region Stockholm, Sweden, with hypertension (Jan 1, 2015 - Feb 28, 2018) of injury and accidents (ICD-10: S00-99: Injuries on body parts, specified, and T00-78: Effects of external injuries) in specialist care, per 1000 females and males.

Supplementary Figure 10: The quarterly cumulative incidence among study participants in Region Stockholm, Sweden, with hypertension (Jan 1, 2015 - Feb 28, 2018) of death due to accidents (ICD-10: S00-99, T00-T78), per 1000 females and males.

Supplementary Figure 11: The quarterly cumulative incidence among study participants in Region Stockholm, Sweden, with hypertension (Jan 1, 2015 - Feb 28, 2018) of death due to suicide (ICD-10: X60-X84), per 100 000 females and males.

Supplementary Figure 12: The quarterly cumulative incidence among study participants in Region Stockholm, Sweden, with hypertension (Jan 1, 2015 - Feb 28, 2018) of death due to events of undetermined intent (such as poisoning, drowning, etc.) (ICD-10: Y10-Y34), per 100 000 females and males.
